# Chemopreventive effects of Ku-jin tea against AOM-induced precancerous colorectal lesions in rats and metabolomic analysis

**DOI:** 10.1038/s41598-017-16237-0

**Published:** 2017-11-21

**Authors:** Wu Bi, Haibo Liu, Jie Shen, Ling-hua Zhang, Pei Li, Bing Peng, Li Cao, Pengfei Zhang, Chunnian He, Peigen Xiao

**Affiliations:** 1 Institute of Medicinal Plant Development, Chinese Academy of Medical Science, Peking Union Medical College, Beijing, 100193 People’s Republic of China; 2 Key Laboratory of Bioactive Substances and Resources Utilization of Chinese Herbal Medicine, Ministry of Education, Beijing, 100193 People’s Republic of China; 3 Key Laboratory of Cancer Proteomics of Chinese Ministry of Health, Xiangya Hospital, Central South University, Changsha, Hunan 410008 People’s Republic of China; 4 PhytoMedix Co. 628 Route 10 West, Suite 10B, Whippany, NJ 07981 USA; 5 Beijing Institute of Traditional Chinese Medicine, Beijing Hospital of Traditional Chinese Medicine Affiliated to Capital Medical University, 100010 Beijing, PR China

## Abstract

Ku-jin tea (KJT) is a health beverage prepared from the leaves of the plant *Acer tataricum* subsp. *ginnala* that has been consumed in some regions of China for thousands of years. KJT contains high levels of anti-inflammatory and antioxidative compounds such as ginnalins, but little is known about the chemopreventive effect of KJT on colon cancer. In this study, we investigated the preventive effects of KJT on colon carcinogenesis using the azoxymethane (AOM)-induced precancerous colorectal lesion model in rats. The results showed that the number of aberrant crypts, aberrant crypt foci (ACF) and crypts/focus in rats of the KJT + AOM group were significantly decreased compared with rats of the AOM group (*p* < 0.01). Further exploration of the prevention mechanism of KJT by UPLC-QTOF/MS-based urinary metabolomics showed that 5 metabolic pathways were modulated, including purine metabolism and amino acid metabolism, in the group with KJT. In addition, the levels of the immunomodulatory cytokines IL-1α and IL-10 were significantly decreased, and the levels of IL-2 in the serum of AOM rats increased after KJT treatment. Our present data suggest that KJT can inhibit AOM-induced colonic ACF formation and might be a useful chemopreventive agent against colorectal carcinogenesis.

## Introduction

Colorectal cancer (CRC) is one of the most common forms of cancer and is the third leading cause of cancer-related death worldwide^1^. The incidence and mortality rates of CRC in China have increased continually in recent years. It was reported that there were 3,763 new CRC cases and 1,911 CRC-related deaths for every 10,000 people in China during 2015^2^. In western countries, CRC also remains the second leading cause of cancer death^3^. In addition, CRC patients at advanced stages have a poor prognosis and a low survival rate^4^. Therefore, increased attention has been focused on CRC prevention. It is a particularly relevant public health task to develop more effective interventions, including using natural products, to prevent and treat this disease. Chemoprevention is becoming increasingly important in CRC treatment. Early interventions against colorectal premalignant lesions with herbal teas, medicinal foods and other natural products are now some of the most promising and cost-effective approaches to prevent CRC in large populations^5,6^.

Tea (the leaves from *Camellia* plants) is one of the most popular beverages in the world; substantial evidence from animal studies and clinical trials supports its effectiveness in the prevention of carcinogenesis and other chronic diseases^7–11^. However, many plants other than *Camellia* have been widely used as tea in various cultures. Recently, non-*Camellia* teas have attracted attention due to their potential for preventing and treating metabolic and inflammatory diseases, as well as cancers. Here, we report *in vivo* preventive effects of Ku-jin tea (KJT) in CRC. KJT is a kind of “Non-*Camellia* tea” prepared from leaves of the plant *Acer tataricum* subsp. *ginnala* that has been commonly used in some Chinese regions for thousands of years^12^. KJT contains high levels of anti-inflammatory and antioxidative compounds such as ginnalins^13^. However, the potential chemopreventive effect of KJT remains unknown. Here, we investigated the modulatory effects of KJT on colon carcinogenesis using the rat azoxymethane (AOM)-induced precancerous colorectal lesion model.

Metabolomic analysis is one of the newly developed approaches to identify biomarkers for diseases and evaluate the effects of drug intervention, as well as to understand metabolic mechanisms^14^. UPLC-QTOF/MS(ultra-performance liquid chromatography/quadrupole time-of-flight mass spectrometry) is one of the widely applied techniques in metabolomics studies due to its high sensitivity and reproducibility^15^. Here, to investigate the metabolic profiles and potential biomarkers in a rat model of precancerous colorectal lesion induced by AOM, UPLC-QTOF/MS based metabolomics was applied. Furthermore, KJT was used as a dietary intervention in the same animal model to gain mechanistic insights into its chemopreventive effects against colorectal carcinogenesis.

## Materials and Methods

### Reagents and materials

Azoxymethane was purchased from Sigma Aldrich (St. Louis, MO, USA). HPLC-grade acetonitrile was purchased from Honeywell Burdick and Jackson (Morristown, NJ, USA) and HPLC-grade methanol was purchased from Fisher Scientific (Atlanta, GA, USA). HPLC-grade formic acid was purchased from CNW Technologies GmbH (Dusseldorf, Germany). Physiological saline (0.9%) was purchased from Shijiazhuang No. 4 Pharmaceutical Co., Ltd. (Shijiazhuang, China). All aqueous solutions were prepared with ultrapure water produced with a Milli-Q system (18.2 MΩ, Millipore, Bedford, MA, USA).

### Ku-jin tea infusion preparation and analysis

Ku-jin tea was purchased from the local store in An Ji, Zhejiang Province, China. Tea infusions were prepared as the ratio of 3 g of tea per 100 mL of hot water. The Ku-jin tea analysis was performed according to our previously published protocol^12^. Briefly, tea infusions were filtered through a 0.22 μm nylon filter membrane (Jinteng experiment equipment Co., Ltd., Tianjin, China) and subjected to UHPLC analysis. A DIONEX Ultimate 3000 UHPLC system (Thermo Scientific, Waltham, MA, USA) equipped with an HPG3400 RS Pump, SRD-3400 degasser, WPS-3000T RS AutoSampler, TCC-3000RS Column Compartment, DAD-3000RS Diode Array Detector and Chromeleon chromatography software package (6.8 version) was used in this study. The contents of the tea were calculated using standard curves of gallotannins, gallic acid, ginnalin A, ginnalin B, ginnalin C and 3,6-di-*O*-galloyl-1,5-anhydro-D-glucitol (all were isolated from the leaves of *Acer tataricum* subsp. *ginnala* in our laboratory).

### Animals and treatment

Thirty healthy male Wistar rats (4–6 weeks old, body weight 65–95 g) were purchased from the Institute of Laboratory Animal Science, Chinese Academy of Medical Sciences (CAMS) and Peking Union Medical College (PUMC) (Beijing, China). The rats were housed in cages for one week to adapt to the environment under controlled conditions with a 12 h light-12 h dark cycle (lights on from 6:00 a.m. to 6:00 p.m.), 40–60% relative humidity and 20–25 °C temperature with commercial diet and water available ad libitum. These rats were then randomly divided into 3 groups (Control, AOM and AOM + KJT, 10 rats/group) and were fed either normal water or experimental water containing KJT for 12 weeks. Following one week of drinking either water or KJT, all rats except the control group were injected with AOM (prepared in 0.9% physiological saline, 15 mg/kg bodyweight, s.c., once weekly for 3 weeks) and the rats in the control group were injected with same volume of 0.9% physiological saline. At 16–17 weeks of age, urine and sera were collected for metabolomic analysis using UPLC-QTOF/MS (ultra-HPLC coupled with time-of-flight mass spectrometry). Rats were sacrificed at the end of the KJT administration, and colorectums were collected for the evaluation of aberrant crypts (AC), aberrant crypt foci (ACF) and crypts/focus. Spleens, thymuses and blood were also taken for immunological analysis.

Animal experiments were approved by the Ethics Committee of the Institute of Medicinal Plant Development, CAMS and PUMC (Beijing, China). All experimental procedures were performed in accordance with relevant guidelines established by the Ethics Committee of the Institute of Medicinal Plant Development, CAMS and PUMC.

### Sample Collection

At the end of the 12th week, each rat was housed in one metabolic cage, and 24 h urine samples were collected and then centrifuged at 12000 rpm (4 °C) for 10 min. The supernatants were stored at −80 °C for the LC-MS analysis.

All the rats were anesthetized, and sera samples were collected. Approximately 10 mL of blood per rat was collected from the orbital vein. Blood samples were left for 12 h at 4 °C, then centrifuged at 3000 rpm (4 °C) for 10 min, and the supernatants were collected and stored at −80 °C until analysis.

After the blood collection, rats were sacrificed and tissues were dissected and weighed. Colonic tissue samples were washed with 0.9% physiological saline, then stored in 10% formalin and used for ACF counting.

### Tissue Sample Preparation for ACF Counting

The entire colon (from cecum to anus) was removed and washed thoroughly with 0.9% physiological saline, cut longitudinally, laid flat on a glass plate, and fixed with 10% buffered formaldehyde solution. The colon was then stained with 0.2% methylene blue for 15 min in saline and the extra methylene blue was washed away with phosphate-buffered saline (pH 7.4); to identify ACF for topographical assessment of the colon, mucosal ACF was counted using a light microscope.

### Sample Preparation for UPLC-TOF/MS Analysis

Urine samples were prepared with minor modifications to a previously published method^16^. Briefly, urine samples were thawed on ice at room temperature before analysis, and 200 μL of each aliquot of urine sample was added into a 1.5 mL tube. Then, 200 μL of methanol was added, and after vortexing for 30 s, the mixture was centrifuged at 10,000 rpm (4 °C) for 10 min. The supernatant was filtered through a 0.22 μm membrane and an aliquot of 5 μL was injected for UPLC/MS analysis.

### Chromatography and Mass spectrometry

The samples were analyzed on a Waters Acquity Ultra Performance LC system (Waters Corporation, Milford, MA, USA) equipped with a BEH SST3 C18 column (2.1 mm × 100 mm, 1.7 μm). The Ultra Performance LC system consisted of a vacuum degasser, a binary pump, an autosampler, a column heater and a diode array detector (DAD) coupled to a QTOF analyzer in a SYNAPT HDMS system (Waters Corporation) and equipped with an ESI interface.

Mobile phase (A) was acidified water (0.1% formic acid) while phase (B) was acetonitrile. The temperature of the column was set to 40 °C. The elution program was as follows: 1% B from 0 to 1 min, 1–12% B from 1 to 3 min, 12–50% B from 3 to 8 min, 50–95% B from 8 to 9 min, 95% B from 9 to 10 min. The injection volume was 5 μL, the flow rate was set at 0.5 mL/min, and UV detection of the compounds was performed from 200 to 400 nm.

Parameters of analysis were set using both the positive and negative ion mode. The parameters were set as the follow: cone and capillary voltage was 40 V and 3,500 V, respectively; cone gas rate was 50 L/h; desolvation gas rate was 900 L/h at a temperature of 350 °C; and the source temperature was 100 °C. The data acquisition rate was 0.15 s. Leucine-enkephalin at a concentration of 0.5 μg/mL with a flow rate of 5 μL/min was used as the lockmass in all analyses (in the positive ion mode [M + H] = 556.2771 and in negative ion mode [M−H]^−^ = 554.2615). Data were acquired in the centroid mode. The mass range from m/z 100–1200 was scanned.

### Assessment of inflammatory cytokine levels in serum

A potential immunomodulatory effect of KJT was evaluated in the rats. Interleukin-1α (IL-1α), IL-2, IL-6, IL-10, tumor necrosis factor (TNF-α), and interferon gamma (IFN-γ) levels in rat serum were measured by a cytometric bead array using Milliplex MAP Rat Cytokine/Chemokine Magnetic Bead Panel (EMD Millipore Corporation, Billerica, MA, USA) on a Luminex 200™ instrument (Luminex Corporation, Austin, TX, USA).

### Data analysis

Mean values of each sample were obtained from three replicates and used for further analysis. The significance of the differences was calculated using Tukey’s multiple range test (*p* < 0.05) and SPSS 13.0 software (SPSS Inc., Chicago, IL, USA).

The acquired raw data from UPLC-QTOFMS were analyzed using the MarkerLynx Applications Manager version 4.1 (Waters, Manchester, UK). This allowed data pretreatment procedures such as peak detection, deconvolution, normalization, alignment and data reduction to give a list of mass and retention times that paired with corresponding intensities for all the detected peaks from each data file in the data set. The main parameters were set as follows: retention time range 1–10 min, mass range 100–1000 amu, mass tolerance 0.01, minimum intensity 1%, mass window 0.05, retention time window 0.20, and noise elimination level 6.

The resulting data were analyzed by partial least squares-discriminate analysis (PLS-DA) and orthogonal partial least squares-discriminant analysis (OPLS-DA) using EZinfo 2.0 software (Umetrics AB, Sweden) after Pareto-scaled procedure. On the basis of variable importance in the projection (VIP) threshold of 1 from the PLS-DA model, a number of the metabolites responsible for the differentiation in the metabolic profiles among AOM-induced precancerous lesion rats and the other two groups could be obtained. In parallel, the metabolites identified by the OPLS-DA model were validated at a univariate level using the S-plots.

## Results

### Gallotannin composition in the Ku-jin tea samples

**Figure 1:**
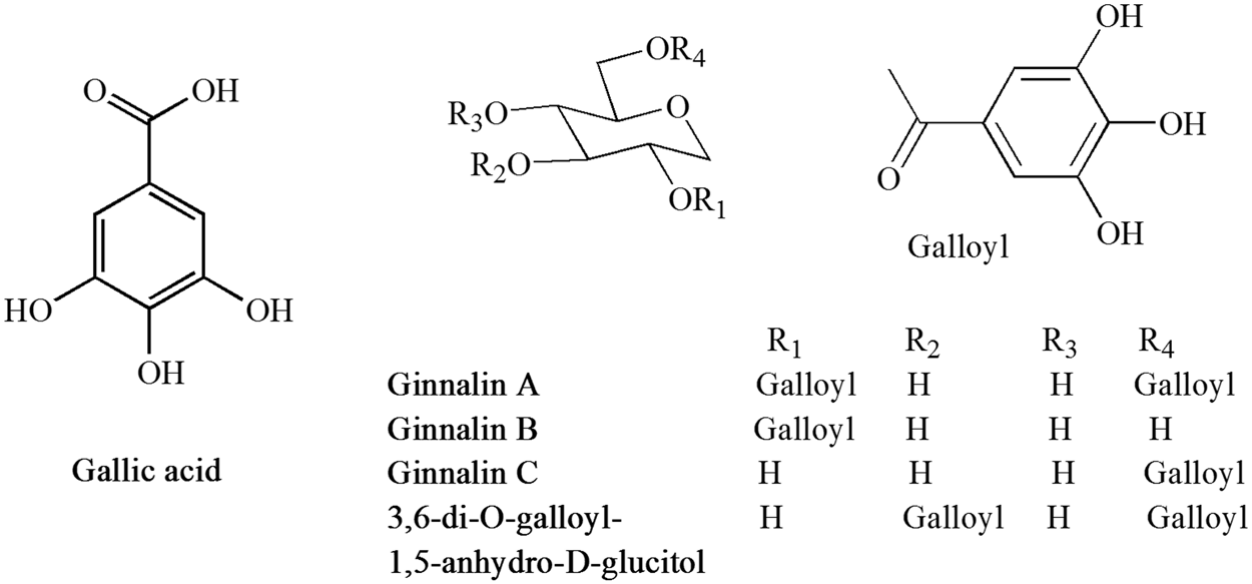
Structure of the main gallotannins in Ku-jin tea.

The metabolite profile and concentrations of the five main gallotannins in Ku-jin tea infusions were measured using UHPLC (Fig. 1 and Supporting Information Figure S1). Content of ginnalin A (187.71 mg/g dry weight) was the highest. Gallic acid (10.23 mg/g dry weight), ginnalin B (23.88 mg/g dry weight) and 3,6-di-*O*-galloyl-1,5-anhydro-D-glucitol (17.92 mg/g dry weight) were found at moderate levels in Ku-jin tea samples. The content of ginnalin C (5.53 mg/g dry weight) was the lowest among all five compounds.

### General observations

**Figure 2:**
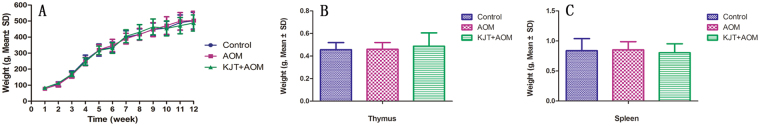
Weight change in the rats (1–12 week) (**A**), weight of the rat spleen (**B**) and thymus (**C**) in different groups (*p* > 0.05, vs Control).

A total of 30 male Wistar rats survived until the end of the experiment, and none of them developed colon tumors. The mean body and immune organ (spleen and thymus) weights for all groups are shown in Fig. 2. No significant effects of AOM and/or KJT treatment on body, spleen and thymus weights were observed.

### Inhibition of AOM-induced precancerous colorectal lesions by Ku-jin tea

**Figure 3:**
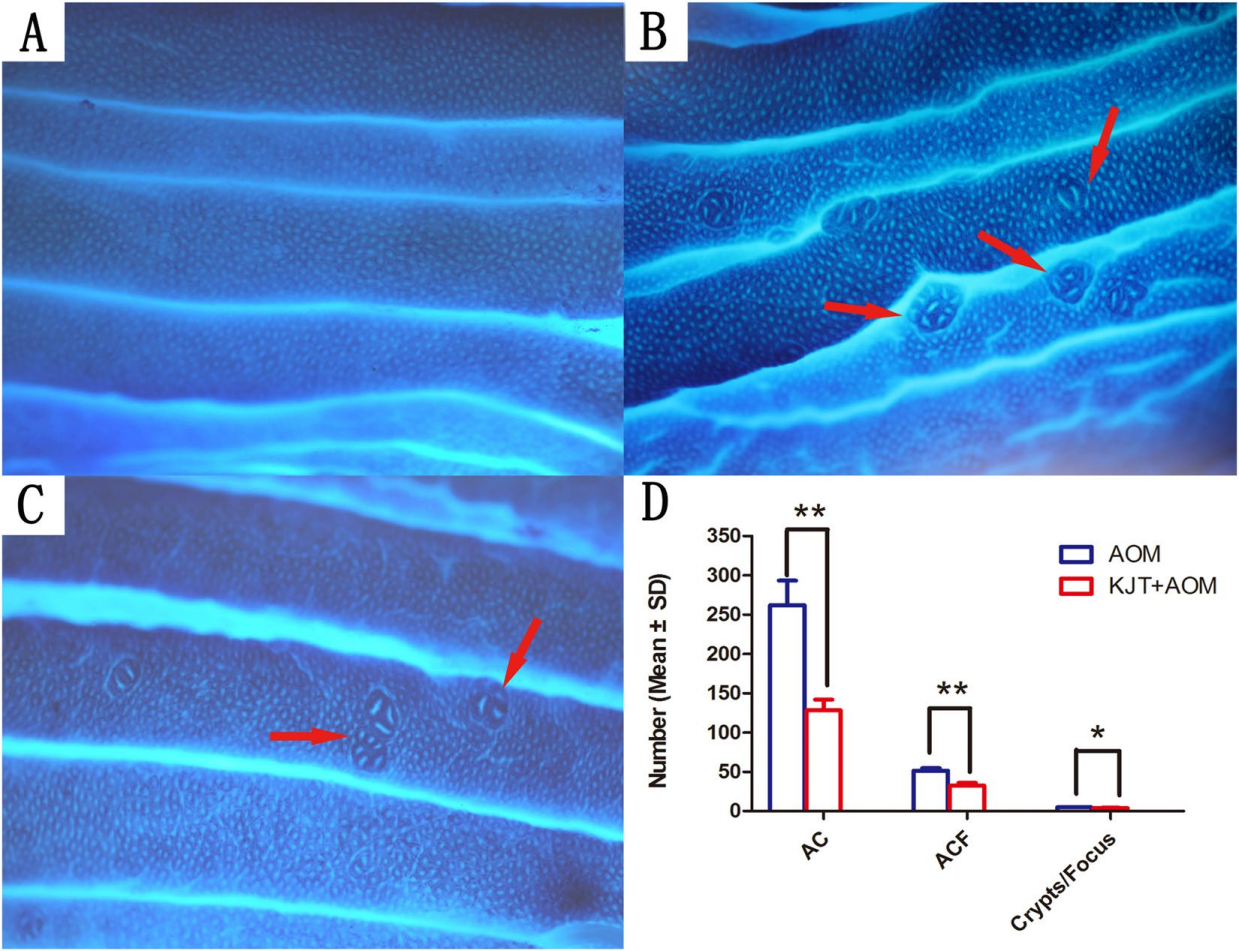
Pathological observation of rat colon tissue. (**A**) Control group; (**B**) AOM group; (**C**) KJT + AOM group; (**D**) Number of AC, ACF and Crypts/Focus (*p < 0.05, **p < 0.01).

Typical histological ACF lesions were found in the AOM group (Fig. 3A,B), which confirms that the precancerous colorectal lesion rat model was successfully produced in the current experiment. A large increase in ACF number was observed in the colon of the AOM group compared with the controls (the number of ACF is 0) at the end of 12th week. Moreover, the number of ACF in the KJT + AOM group were significantly lower than in the AOM group (Fig. 3C,D and Table 1).

**Table 1:** Effect of KJT on rat ACF induced by AOM (Mean ± SD) (*p < 0.05, **p < 0.01).

Group	Incidence of ACF	Number of ACF	Number of AC	Mean number of Crypts/Focus
Control group	1/10	0	0	0
AOM group	10/10	51.4 ± 17.6	262.1 ± 230.9	4.76 ± 1.26
KJT + AOM group	10/10	32.6 ± 21.4**	128.3 ± 95.7**	3.96 ± 0.79*

### Immunomodulatory effects of Ku-jin tea

**Figure 4:**
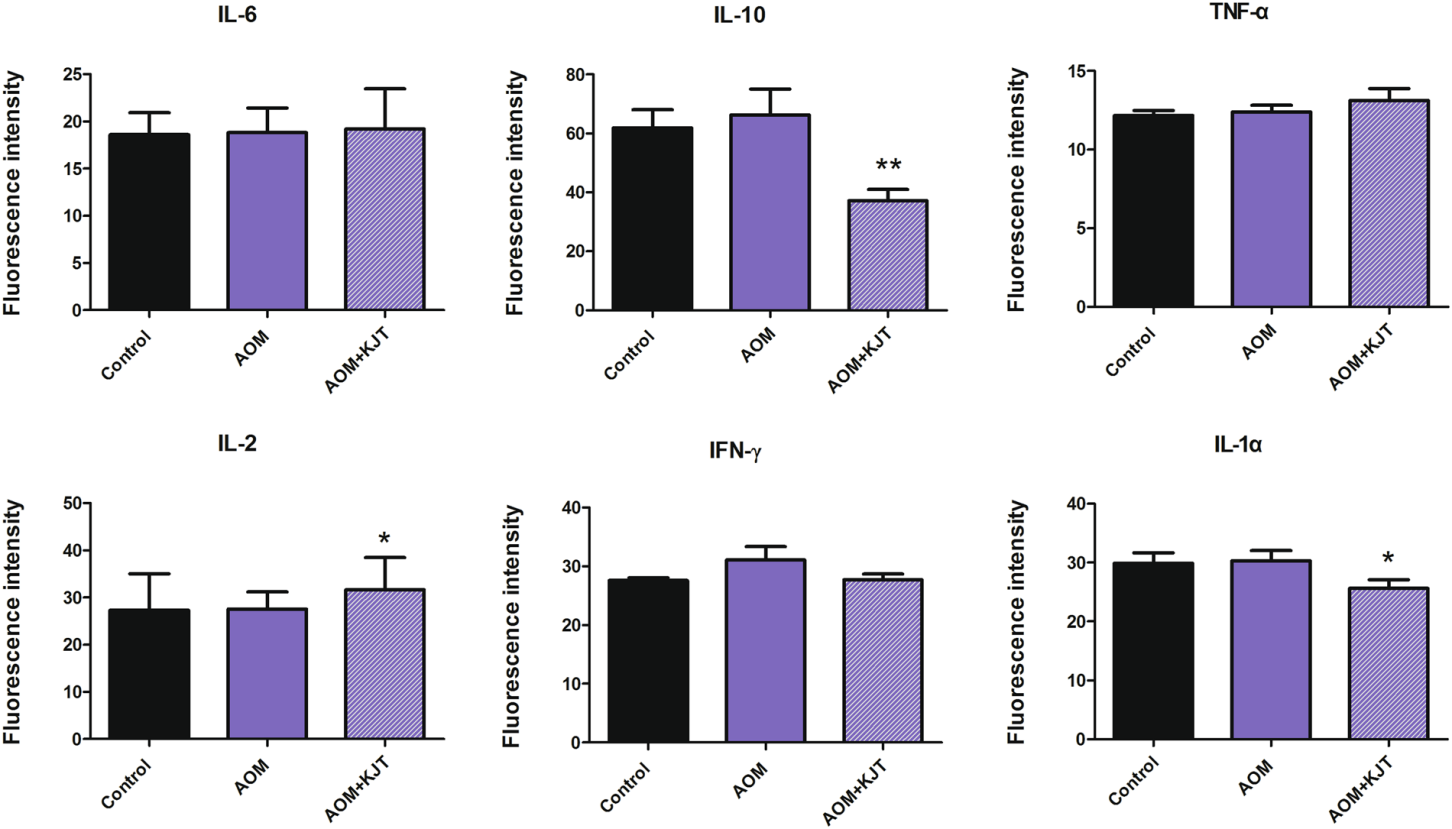
Effect of KJT on cytokine levels in rat serum (*p < 0.05, **p < 0.01, vs AOM group).

To evaluate any potential immunomodulatory activities of KJT on these rats, several critical cytokines involved in the immune response of T lymphocytes and monocytes were tested using serum samples. As shown in Fig. 4, AOM appeared to have no appreciable effect on cytokine production. However, KJT could significantly reduce the levels of IL-1α and IL-10 but enhance the production of IL-2, suggesting that KJT could modulate the immune response of these rats. These data also indicated that KJT might be capable of activating T cell immunity to prevent tumor cell growth.

### Metabolomic analysis of the potential mechanism of the preventive effect of Ku-jin tea on AOM-induced precancerous colorectal cancer

**Figure 5:**
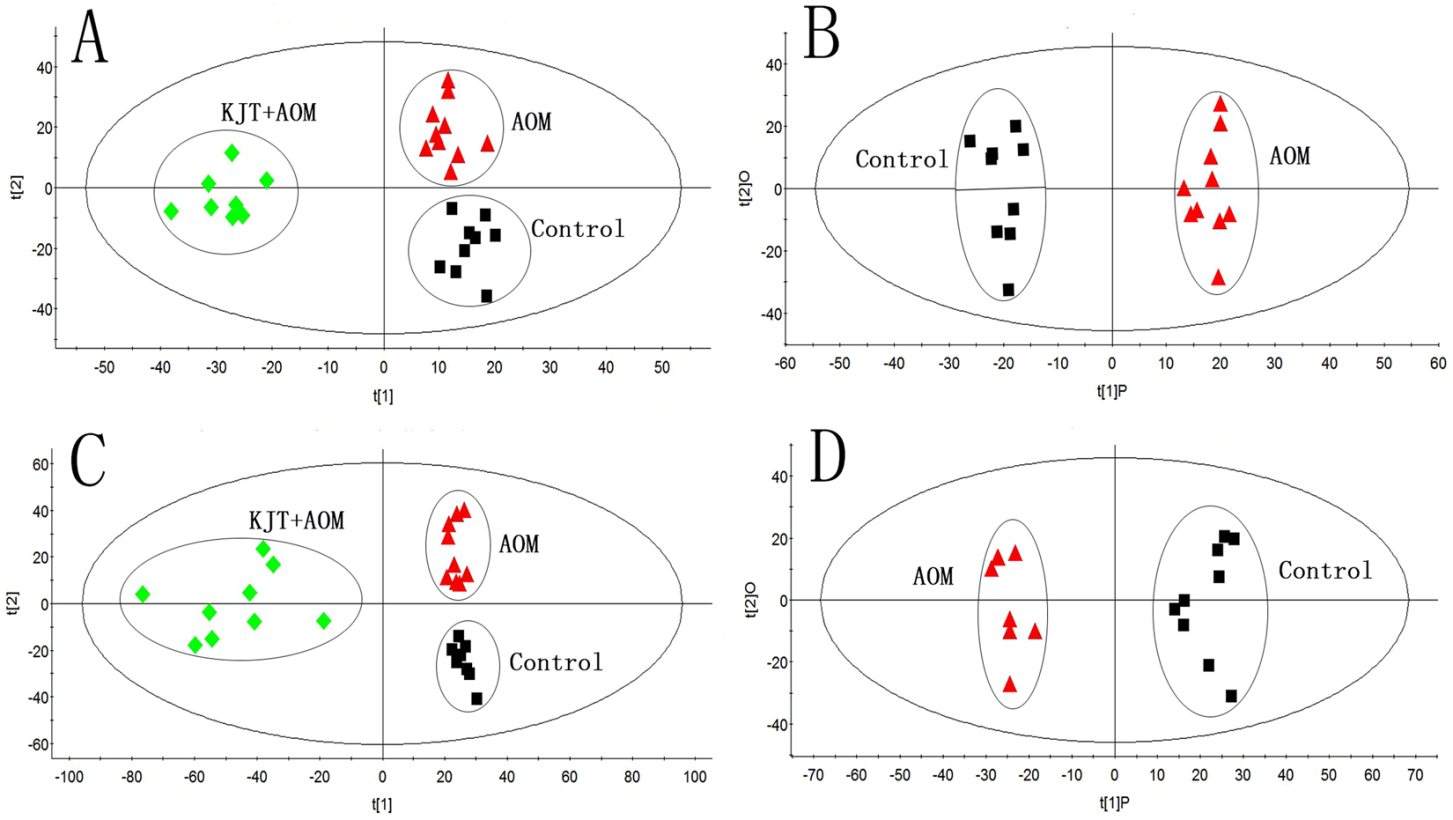
PLS-DA score plots based on the urine metabolite profiling in positive and negative ion modes of the control group, AOM group and KJT + AOM group, respectively (**A**,**C**), and OPLS-DA score plots between the control group and AOM group, respectively (**B**,**D**). The AOM group and control group were clearly separated, indicating that the urinary metabolic pattern was significantly changed with the process of AOM.

To evaluate the effect of KJT on the rats and to explore the targeted pathways, a metabolomics analysis of animal urine samples was performed. Figure 5 illustrates the score plots of PLS-DA and OPLS-DA model of the subjects from the control group, AOM group and KJT + AOM group based on the urine metabolites of rats. The KJT + AOM group and the AOM group were both clearly separated from the controls. The PLS-DA score plots constructed with all the LC-TOF/MS spectral features from urine of the control group, AOM group, and KJT + AOM group are provided in Supporting Information Figure S2.

**Figure 6:**
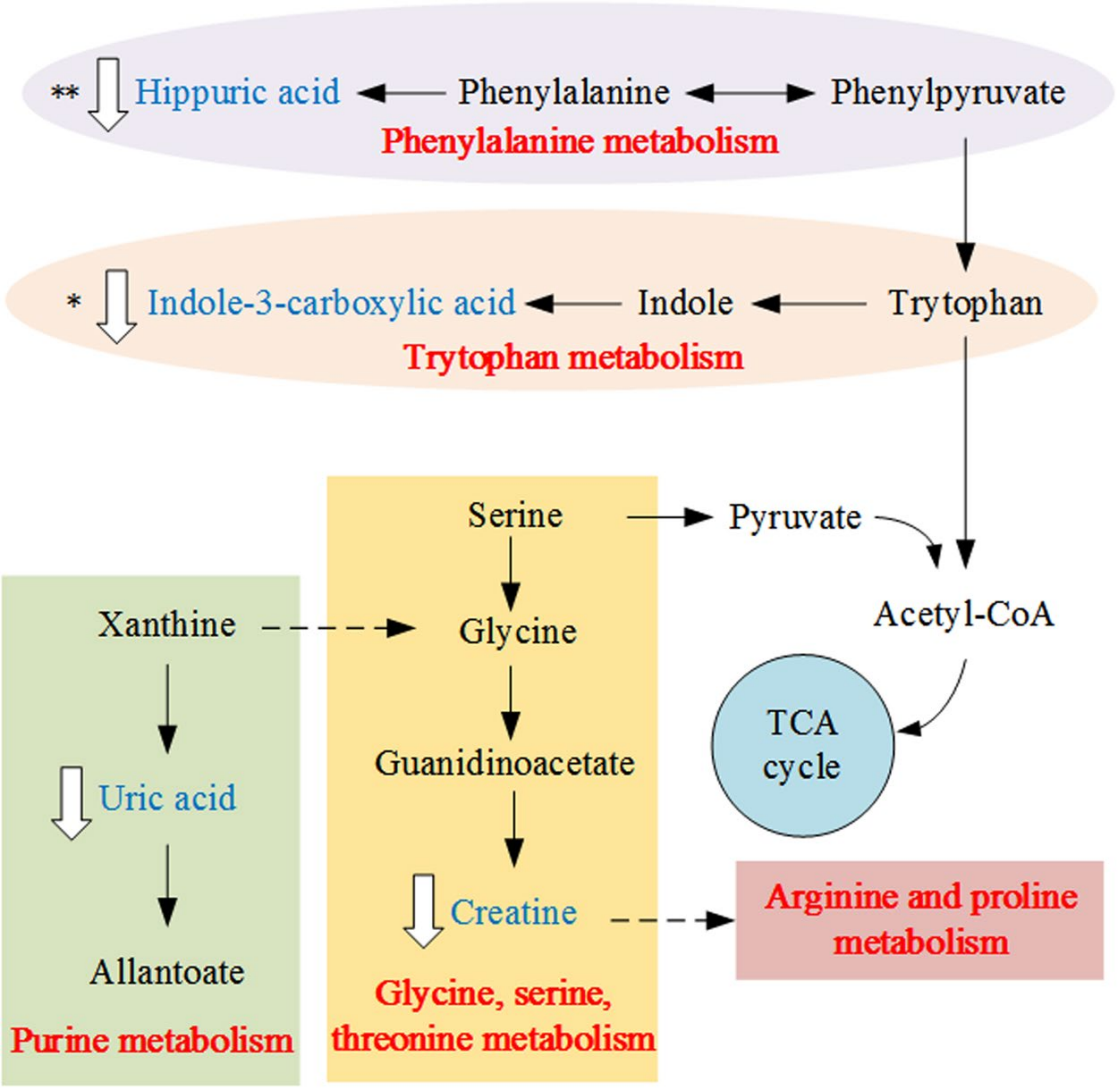
Potential metabolic pathways related to AOM-induced colorectal cancer.

A total of 13 differentially expressed urine metabolites were selected as potential biomarkers according to the VIP values (VIP > 1) and S-plots in the OPLS-DA models, and 9 of them were tentatively identified by analyzing their accurate molecular weights and MS/MS spectra. Databases, such as HMDB (http://www.hmdb.ca/), Metlin (http://metlin.scripps.edu), and KEGG (http://www.kegg.jp/), were used for confirmation. Compared with controls the altered metabolites included increased 9-decenoylcarnitine (**3**) and octanoyl glucuronide (**11**) and decreased creatine (**1**), 5-methyldeoxycytidine (**2**), uric acid (**4**), indole-3-carboxylic acid (**5**), hippuric acid (**9**) and *p*-cresol glucuronide (**10**) in the urine of AOM group rats. The variation in differential metabolites listed in Table 2 upon AOM intervention were also investigated in the KJT + AOM group, and as a result, the changing trend of all the metabolites were partially reversed after KJT treatment. Through KEGG and literature searches, those altered metabolites were determined to be related to 5 metabolic pathways including purine metabolism; glycine, serine and threonine metabolism; arginine and proline metabolism; phenylalanine metabolism; and tryptophan metabolism (Fig. 6) and were also associated with the gut microbiota. Therefore, our results indicated that those metabolic pathways were disturbed in the urine of AOM-induced colon cancer rats. In addition, these disturbances could be rectified by KJT treatment. Therefore, the potential mechanism underlying the preventive effect of KJT on AOM-induced precancerous colorectal cancer was perhaps associated with these five metabolic pathways.

**Table 2:** Potential biomarkers and the trends associated with precancerous lesions in colon cancer (“↑”, increase in signal; “↓”, decrease in signal, *p < 0.05, **p < 0.01).

No.	Retention time (min)	QTOF (m/z)	Ion	VIP Value	Metabolites	Proposed Molecular Formula	AOM group/Control group	KJT + AOM group/AOM group	Metabolite pathway
**1**	0.55	132.0771	[M + H]^−^	11.59	Creatine	C_4_H_9_N_3_O_2_	↓	↑**	Glycine, serine and threonine metabolism, arginine and proline metabolism
**2**	1.49	242.1131	[M + H]^−^	4.52	5-Methyldeoxycytidine	C_10_H_15_N_3_O_4_	↓**	—	—
**3**	6.96	314.2323	[M + H]^−^	4.23	9-Decenoylcarnitine	C_17_H_31_NO_4_	↑*	↓	—
**4**	1.00	169.0362	[M + H]^−^	5.55	Uric acid	C_5_H_4_N_4_O_3_	↓	↑	Purine metabolism, oxidative damage
**5**	4.50	162.0548	[M + H]^−^	8.92	Indole-3-carboxylic acid	C_9_H_7_NO_2_	↓*	↑	Tryptophan metabolism
**6**	4.44	368.1547	[M + H]^−^	6.96	unknown	—	↑**	↓*	—
**7**	6.76	380.1913	[M + H]^−^	6.04	unknown	—	↑**	↓	—
**8**	4.44	349.1132	[M-H]^−^	14.03	unknown	—	↑**	↓*	—
**9**	4.12	178.0499	[M-H]^−^	12.21	Hippuric acid	C_9_H_9_NO_3_	↓**	↑**	Gut microbiota
**10**	4.78	283.0816	[M-H]^−^	13.11	*p*-Cresol glucuronide	C_13_H_16_O_7_	↓*	↑	Gut microbiota
**11**	6.57	319.1394	[M-H]^−^	9.73	Octanoyl glucuronide	C_14_H_24_O_8_	↑**	↓	—
**12**	4.54	335.1338	[M-H]^−^	9.29	unknown	—	↑**	↓**	—
**13**	5.56	363.1648	[M-H]^−^	7.47	unknown	—	↑**	↓	—

## Discussion

Chemoprevention of chronic diseases such as cancers through food or drink has gained significant attention in recent years due to the increase in the cost of medical treatment. The prevention of chronic diseases by various foods or drinks represents an inexpensive, readily applicable approach to control and reduce cancer incidence. Foods and drinks are comprised of many beneficial nutrients, especially natural dietary compounds, that together may have additive or synergistic properties to reduce cancer risk. A great number of studies in recent years demonstrated that not only *Camellia* tea (such as green tea, black tea) but also non-*Camellia* teas (such as Sage Tea, Yerba mate tea, or African Rooibos tea) have preventive effects on colon cancer^5,17–20^.

Recently, ginnalin A, ginnalin B and ginnalin C from two endemic North American maple species (*Acer saccharum* Marsh and *A. rubrum* L.) have been reported to have potential colon cancer chemopreventive agents *in vitro* ^21–23^, but *in vivo* evidence is lacking. Furthermore, the compounds ginnalin A, ginnalin B and ginnalin C were first isolated from Ku-jin tea^13^; the antitumor effects of Ku-jin tea have not been studied until now. In the present study, we demonstrated for the first time the potential chemopreventive effect of Ku-jin tea on colon cancer *in vivo*.

Aberrant crypt foci (ACF) are visible preneoplastic lesions and are identified as putative precursors to colon cancer in animals and humans^24–26^. Thus, ACF have been widely used as a useful biomarker for colon carcinogenesis, and inhibition of the development of ACF can be used to assess anticancer activity^27–29^. Due to similarities with the pathophysiology of human colon tumors, the AOM-induced model is a very useful laboratory method to find preventive agents for CRC and to explore the potential molecular mechanisms involved in the initial stage of CRC^30^. In our present results, all the rats in the KJT + AOM group showed significantly less numbers of ACF compared to the AOM group, demonstrating the preventive effects of KJT on the AOM-induced precancerous colorectal lesions.

Inflammation plays a critical role in carcinogenesis. It was estimated by an epidemiological study that up to 40% of patients with colitis developed colitis-associated CRC^31^. In colonic tumorigenesis, the inflammatory cells contributed to the colitis by generating proinflammatory cytokines, such as IL-1α, IL-2, IL-6, TNF-α, INF-γ. Since inflammation is closely linked to tumor formation, substances with potent anti-inflammatory activities are anticipated to exert chemopreventive effects against carcinogenesis, particularly during the developmental stage^32^. Therefore, in the current investigation, inflammatory cytokines secreted into blood were examined in order to explore the anti-inflammatory properties of KJT. We found that KJT significantly decreased the concentration of IL-1α and IL-10 and stimulated IL-2 production but had no obvious effect on the other three cytokines (IL-6, TNF-α, INF-γ). Our findings provide the first molecular evidence that KJT can enhance rat immunity. IL-1α and IL-10 were significantly increased and IL-2 were significantly decreased in patients with colon cancer^33–35^, thus demonstrating the anti-inflammatory activity and immune-enhancing effect of KJT. IL-1α, as an important proinflammatory cytokine in the tumor microenvironment, can regulate tumor progression by increasing tumor growth, angiogenesis, and invasiveness^36,37^. Previous studies have shown that down regulating the expression of IL-1α could inhibit or attenuate cancer progression, especially for colorectal cancer^37,38^. IL-10 is not only produced by various immunocompetent cells but also by human cell lines derived from carcinomas such as colon, kidney, and breast. Although IL-10 is an anti-inflammatory cytokine and exhibits various immunosuppressive effects *in vivo*, it has been demonstrated that some colon cancer cells (for example, HT-29) can produce IL-10 themselves, resulting in tumors escaping the immune system’s defenses^39,40^. Interestingly, our results indicated that KJT has the potential to reduce IL-10 levels in rats, thus suggesting a lower capacity to escape the immune system. IL-2 is also an important immunoregulatory factor and plays multiple roles in the host immune system. It was the first FDA-approved cytokine for cancer therapy, displaying remarkable antitumor potential^41^. It is suggested that the anti-CRC effect of KJT may be through increased IL-2 levels.

To date, mechanistic studies aimed at understanding the metabolic alterations in cancer cells from Ku-jin tea that lead to cancer prevention have not been reported. Metabolomic analysis is a valuable tool for detecting metabolic alterations. The results from urine metabolomics indicated that Ku-jin tea could intervene in some pathways of amino acids and energy metabolism. A previous study reported that metabolites mainly involved in energy and amino acid metabolism were changed in patients with CRC^42^. For example, creatine (**1**), 5-methyldeoxycytidine (**2**) and indole-3-carboxylic acid (**5**) were related to amino acid metabolism. Creatine levels in animals are synthesized *de novo* from the liver via the use of amino acids, such as arginine, glycine, and methionine. In addition, creatine is usually excreted as creatinine into the urine and provides energy to muscles in the form of stored creatine phosphate^43^. Loss of DNA 5-methyldeoxycytidine residues in old age can disrupt the cellular gene expression and contribute to the physiological decline of the animal^44^. Indole-3-carboxylic acid is the metabolite of tryptophan, the precursor of the neurotransmitter 5-hydroxy tryptamine^45^. The level of indole-3-carboxylic acid was significantly increased in AOM-induced rats compared with control rats. Uric acid (**4**) is related to energy metabolism. It is the final oxidation product in purine metabolism and is produced by the enzyme xanthine oxidase, which oxidizes oxypurines such as xanthine into uric acid^46^. Additionally, hippuric acid (**9**) and *p*-cresol glucuronide (**10**) are involved in gut flora metabolism and were also found to be significantly altered in the urine of the AOM group compared to the control group. There have been several studies reporting the metabolite changes involved in the microbial metabolism of CRC animals and patients^6,47,48^. *p*-Cresol glucuronide, which is a uremic toxin, is a derivative of *p*-cresol excreted in the urine. *p*-Cresol is mainly generated from tyrosine or phenylalanine by anaerobic intestinal bacteria^49^. Decreased excretion of *p*-cresol glucuronide found in the rats may have been due to the disturbance of the normal microbial ecosystem in the presence of AOM. Hippuric acid is a metabolite of phenylalanine that is produced by the gut microflora. Urinary hippuric acid was found to be a biomarker of early kidney injury or acute kidney injury in previous metabolomic studies in rats^50^.

In summary, Ku-jin tea significantly inhibited AOM-induced colonic ACF formation, suggesting that KJT might have a chemopreventive effect against colon carcinogenesis, at least in the initial stage of CRC. Furthermore, KJT may exert chemopreventive effects on ACF formation by changing metabolic pathways and by modulating immune and inflammation responses. Plant foods and beverages, such as Ku-jin tea, have shown great potential in the prevention of colon cancer, and they should be considered as potential chemopreventive agents in dietary strategies against colon cancer.

## Electronic supplementary material

Supplementary materials
