# Supplementary material for: Chemopreventive effects of Ku-jin tea against AOM-induced precancerous colorectal lesions in rats and metabolomic analysis
Source: Sci Rep. 2017 Nov 21;7:15893. doi: 10.1038/s41598-017-16237-0 (PMC5698479; doi:10.1038/s41598-017-16237-0)
Supplement: Supplementary file 1 — Supplementary materials [file 41598_2017_16237_MOESM1_ESM.docx]

**Chemopreventive effects of Ku-jin tea against AOM-induced precancerous colorectal lesions in rats and metabolomic analysis**

**Wu Bi^a,b,c^, Haibo Liu^a,b^, Jie Shen^a,b^, Ling-hua Zhang^d^, Pei Li^a,b^, Bing Peng^e^, Li Cao^a,b^,** **Pengfei Zhang^c^, Chunnian He^a,b^*, Peigen Xiao^a,b^**

^a^*Institute of Medicinal Plant Development, Chinese Academy of Medical Science, Peking Union Medical College, Beijing, 100193, People's Republic of China*

^b^*Key Laboratory of Bioactive Substances and Resources Utilization of Chinese Herbal Medicine, Ministry of Education, Beijing, 100193, People's Republic of China*

*^c^Key Laboratory of Cancer Proteomics of Chinese Ministry of Health, Xiangya Hospital, Central South University, Changsha, Hunan 410008, People’s Republic of China*

^d^*PhytoMedix Co. 628 Route 10 West, Suite 10B, Whippany, NJ 07981, USA*

^e^*Beijing Institute of Traditional Chinese Medicine, Beijing Hospital of Traditional Chinese Medicine Affiliated to Capital Medical University, 100010 Beijing, PR China*

* Corresponding author; Tel.: +86-10-57833165; Fax: +86-10-57833166; E-mail: cnhe@implad.ac.cn

**Supplementary materials**


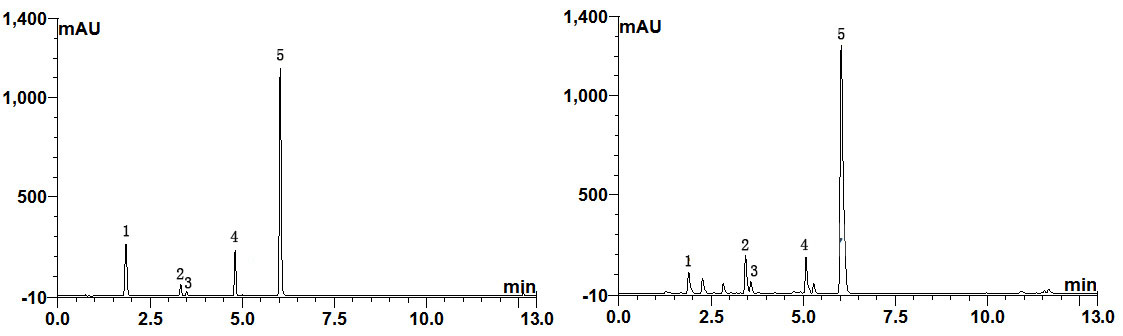


Figure S1 The chromatogram of ginnalins in Kujin tea. A.UHPLC peak assignments of the five ginnalins in standard solution: 1, Gallic acid; 2, Ginnalin B; 3, Ginnalin C; 4, 3,6-di-O-galloyl-1,5-anhydro-D-glucitol; 5, Ginnalin A. B. The five ginnalins in tea infusions.


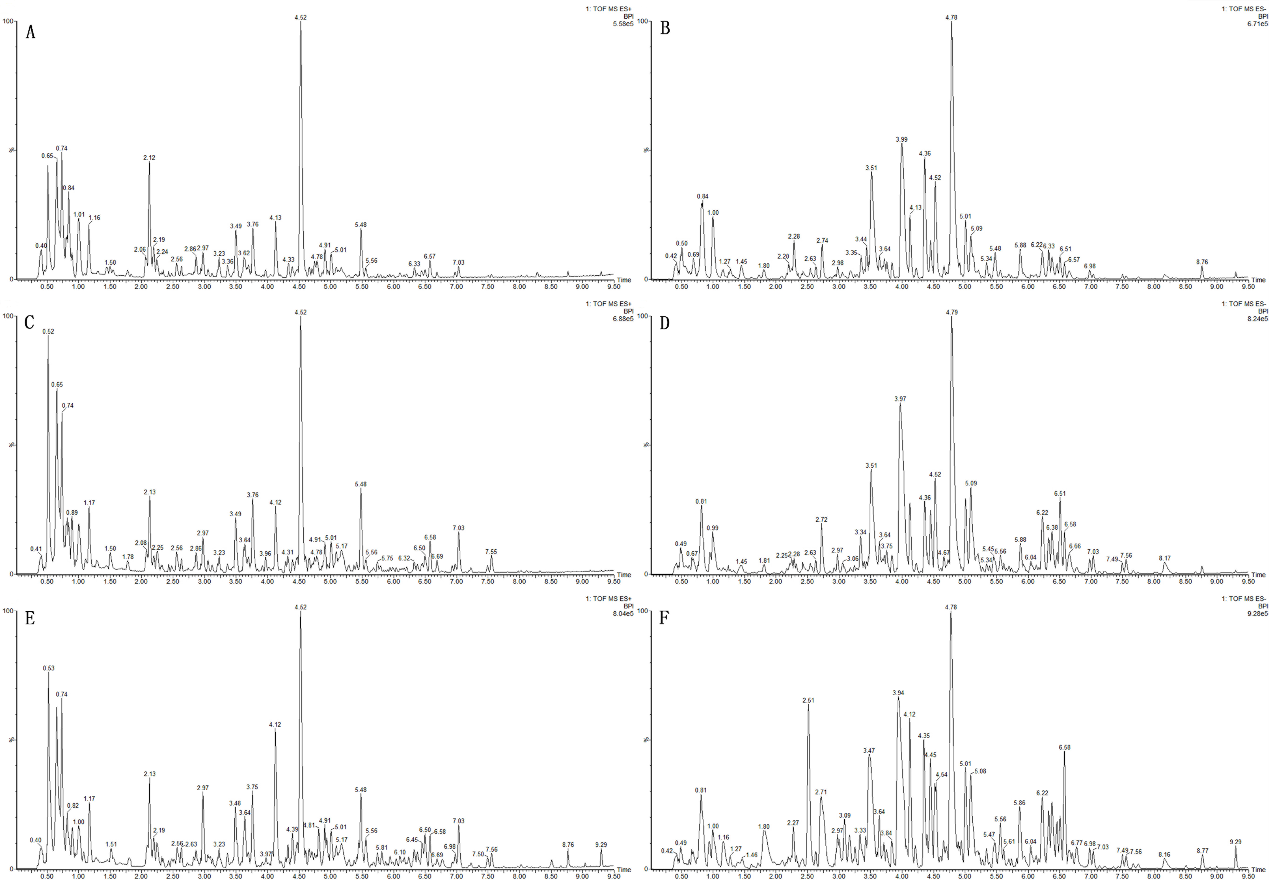


Figure S2 The UPLC/MS positive and negative total ion chromatogram of Control group (A, B), AOM group(C, D), and KJT+AOM group (E, F).


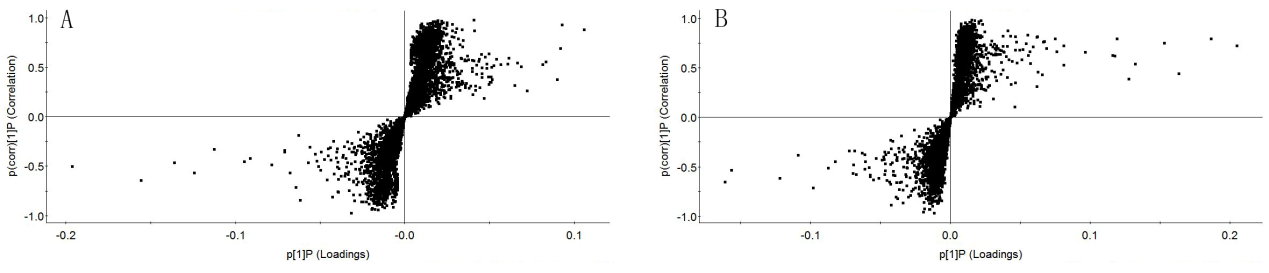


Figure S3 S-plot of OPLS-DA for urine samples of AOM-group *versus* Control group at positive ion mode (A) and negative ion mode (B), respectively.
